# [^18^F]Fluorocholine PET/CT-guided stereotactic body radiotherapy in patients with recurrent oligometastatic prostate cancer

**DOI:** 10.1007/s00259-019-04482-6

**Published:** 2019-10-16

**Authors:** Francesco Pasqualetti, Marco Panichi, Martina Sollini, Aldo Sainato, Luca Galli, Riccardo Morganti, Serena Chiacchio, Andrea Marciano, Roberta Zanca, Lorenzo Mannelli, Gabriele Coraggio, Andrea Sbrana, Paola Cocuzza, Sabrina Montrone, Davide Baldaccini, Alessandra Gonnelli, Alessandro Molinari, Martina Cantarella, Valentina Mazzotti, Sergio Ricci, Fabiola Paiar, Paola Anna Erba

**Affiliations:** 1 Radiation Oncology, Pisa University Hospital, Via Roma 67, 56123 Pisa, Italy; 2 Department of Biomedical Sciences, Humanitas University, Via Rita Levi Montalcini 4, Pieve Emanuele, Milan, Italy; 3 Medical Oncology, Pisa University Hospital, Via Roma 67, 56123 Pisa, Italy; 4 Statistic Analysis Unit, Pisa University Hospital, Via Roma 67, 56123 Pisa, Italy; 5 Nuclear Medicine, Pisa University Hospital, Via Roma 67, 56123 Pisa, Italy; 6 IRCCS SDN Naples, Naples, Italy; 7 Radiation Oncology, Hopitaux Universitaires Henri Mondor APHP, Créteil, France; 8 Radiation Oncology, Casa di Cura SanRossore, Pisa, Italy

**Keywords:** Prostate cancer, [^18^F]FMCH PET/CT, Stereotactic body radiotherapy

## Abstract

**Background:**

In the last years, functional imaging has given a significant contribution to the clinical decision-making of biochemically relapsed prostate cancer (PCa). Hereby, we present a prospective study aiming to validate the role of [^18^F]Fluoro-Methyl Choline ([^18^F]FMCH) PET/CT in the selection of PCa patients suitable for stereotactic body radiotherapy (SBRT).

**Methods:**

Patients with biochemical recurrence limited up to three lesions revealed by [^18^F]FMCH PET/CT were enrolled in the present study and treated with SBRT on all active lesions. Systemic therapy-free survival since the [^18^F]FMCH PET/CT was considered as the primary endpoint.

**Results:**

Forty-six patients were evaluated, and a total of 67 lesions were treated. After a median follow-up of 28.9 months, systemic therapy was started in 30 patients (65.2%) and median systemic therapy-free survival was 39.1 months (95% CI 6.5–68.6); 6, 12, and 24-month ratios were 93.5%, 73.9%, and 63.1%, respectively. At univariate Cox regression analysis, Delta PSA demonstrated an impact on systemic therapy-free survival (*p* < 0.001).

**Conclusions:**

Based on our findings, [^18^F]FMCH PET/CT can identify oligometastatic prostate cancer patients suitable for SBRT, resulting in a systemic therapy-free survival of 39.1 months.

## Background

Prostate cancer (PCa) is the most common malignant tumor in men in Western countries and, until recently, the metastatic disease has been considered as a unique clinical entity requiring up-front systemic therapy, either hormonal or chemotherapy [1, 2].

In the last years, functional imaging has given a significant contribution to clinical decision-making of biochemical relapsed PCa, allowing early diagnosis of metastatic disease [3, 4]. The extensive use of functional imaging resulted in the identification of a new clinical disease entity called oligometastatic disease and characterized by a limited tumor burden [5, 6]. Consequently, the paradigm of metastatic PCa has evolved and, nowadays, tumor burden is a crucial parameter that must be considered in the treatment planning of selected patients [5, 7–11]. Several experiences have even shown that by treating with local ablative approaches, such as stereotactic body radiotherapy (SBRT), all the lesions revealed by functional imaging, a PSA fall can be achieved [5, 7, 8]. Moreover, the demonstration of PSA control with repeated SBRT has led several authors to investigate the administration of systemic therapy only in case of PSA failure due to diffuse disease, a condition not suitable for SBRT. However, currently, most of the studies evaluating SBRT in oligometastatic disease have a retrospective design and are characterized by a pure radiotherapy focus.

Hereby, we present a prospective study aiming to validate the role of [^18^F]Fluoro-Methyl Choline ([^18^F]FMCH) PET/CT in the selection of PCa patients suitable for SBRT.

## Methods

### Study design

The present prospective study was approved by the local Ethics Committee (Pisa 2015/8424); data were collected prospectively, and interventional procedure was not performed. Accrual was completed in June 2018, and data analysis was performed in November 2018. Patients with biochemical recurrence after first-line curative treatments failure were screened. Biochemical relapse was defined as follows: two consecutive PSA values > 0.2 ng/mL in patients underwent radical prostatectomy, three successive increases in PSA value following PSA nadir after primary external beam radiotherapy (EBRT), three consecutive PSA values demonstrating therapy failure and testosterone level < 50 ng/mL in castration-resistant PCa (CRPC). Both hormone-sensitive and castration-resistant patients were considered. Eligible patients were imaged with endo-rectal magnetic resonance to exclude local recurrence inside the prostate bed and [^18^F]FMCH PET/CT to assess tumor burden. Patients with up to three synchronous active lesions identified by [^18^F]FMCH PET/CT were enrolled in the present study. All patients enrolled were treated with SBRT on all active lesions revealed by [^18^F]FMCH PET/CT and signed a consent for the study before SBRT.

### Choline PET/CT

#### Imaging protocol

PET/CT images were acquired 45–60 min after [^18^F]FMCH (IASOcholine® from IASON, Graz, Austria) i.v. administration (about 4 MBq/kg of body weight) using a GE Discovery 710 ST scanner (GE Healthcare). Images were analyzed using a dedicated PET/CT review software (Advantage for Windows, versions 4.2 to7; GE Medical Systems). PET/CT images were interpreted by three nuclear medicine specialists (PAE, MS, and LM) who were aware of the patient’s medical history and PSA values. [^18^F]FMCH PET/CT images were interpreted as positive in the presence of at least one area of abnormal [^18^F]FMCH uptake.

#### Imaging interpretation

According to the number of the lesions detected by [^18^F]FMCH PET/CT, patients were classified on oligometastatic (< 4 lesions) or plurimetastatic (≥ 4 lesions). Additionally, lesions were also classified according to the anatomical site in local recurrence (i.e., lodge), regional recurrence (i.e., the involvement of the lymph nodes below the common iliac artery), distant recurrence (i.e., any other lymph-node chain), and bone disease.

### Stereotactic body radiotherapy

SBRT treatment was performed using a Varian True Beam® platform and 6-MV photons with flattening filter-free beams. RapidArc®system was used for treatment planning. Clinical target volume (CTV) was defined using [^18^F]FMCH-PET/CT scan imaging. Planning target volume consisted of both bone and nodal lesions of an isotropic 3-mm expansion of the CTV. Two different schedules of radiotherapy were considered: 24 Gy as a single fraction or 27 Gy in three fractions, 2–3 sessions a week. Dexamethasone (4 mg) was administered 1 h after the SBRT session in the patients treated with 24 Gy. Task group 101 of the American Association of Physicists in Medicine constraints were used to avoid the toxicity of the organs at risk [12]. Toxicity was recorded using the Common Terminology Criteria for Adverse Events (CTCAE) version 4.0.

### Patients’ follow-up

After SBRT, patients were followed-up with total PSA determination at 6 week and every 3 months in the first 2 years of the study, then every 6 months. In the presence of biochemical recurrence following SBRT (two consecutive rises measured over 6 weeks), a new [^18^F]FMCH-PET/CT scan was performed. If an oligometastatic disease still persisted, further SBRT was planned, otherwise, when more than three active synchronous lesions were detected, patients were treated with systemic treatment in accordance with European Association of Urology (EAU) Guideline, even though in case of asymptomatic disease [2].

### Statistical analyses

Descriptive statistics reports patients’ main characteristics at the time of study enrollment. Survival probability without systemic therapy (systemic therapy-free survival) was estimated by the Kaplan-Meier method. Univariate Mantel-Cox analysis was used to examine the predictive value of covariates. All *p* values were set at 0.05. Systemic therapy-free survival was considered as the primary endpoint. The number of treated lesions, time to relapse after local curative therapy, presence of bone or nodal metastatic disease, the decline between baseline PSA pre-SBRT and PSA value 6 weeks after treatment (ΔPSA), PSA doubling time, Gleason Score, and castration-resistant disease (yes or no) were considered as covariate (Table 2). Cases were censored either at the beginning of systemic therapy or during the last follow-up visit if no systemic therapy was administered.

Statistical analysis was performed with SPSS v.15.0 (IBM Corp, Somers, NY, USA).

## Results

Fifty-one patients were included in the present study. Forty-six patients with oligometastatic PCa (castration sensitive in 35 patients, castration-resistant in 11) for a total of 67 lesions (lymph node and bone lesions, in 45 and 22 cases, respectively) were considered evaluable for the present analysis. Among the 44 patients who underwent radical prostatectomy, pT2 and pT3 stages were recorded in 11 and 33 patients, respectively; 11 of these patients presented even a nodal involvement.

After SBRT, five patients were lost at follow-up since they started ADT for a personal reason. Patients’ median age at the time of study entry was 70 years (range 50–81). The median length between PCa diagnosis and study enrollment was 69 months (range, 2–180 months). At the time of study entry, Median PSA value was 2.69 ng/mL (range 0.9–27.40). Table 1 shows baseline patients’ characteristics. After the first SBRT, two patients developed a failure in the prostate bed. They were both treated with salvage external beam radiotherapy and, because the achievement of PSA control, they were still considered oligometastatic and appropriate for this study.

**Table 1:** Patient characteristics

	Value
Number of patients	46
At primary diagnosis	
Age at diagnosis (years)	
Median	70.13
Range	59–81
Gleason score	
Median	7
Range	5–9
Follow-up from PCa diagnosis (Months)	
Median	22.3
Range	4.3–88.4
PSA at study entry (ng/mL)	
Median	2.69
Range	0.04–27.40
PSA DT (months)	
Median	12
Range	1.6–17.4
Treatments at diagnosis	
Radical prostatectomy alone	17 (35.5%)
Radical prostatectomy with postoperative RT	27 (58.9%)
Radiotherapy alone	2 (4.2%)
At SBRT	
Site of lesions, *n* (%)	
Bones	22 (32.9%)
Lymph nodes	45 (67.1%)
Number of lesions treated at first recurrence	(Total 46)
1 lesion	39(84.8%)
2 lesions	6 (13.0%)
3 lesions	1 (2.2%)
Systemic therapy	
No	21 (46.6%)
Yes	24 (53.3%)

**Fig. 1:**
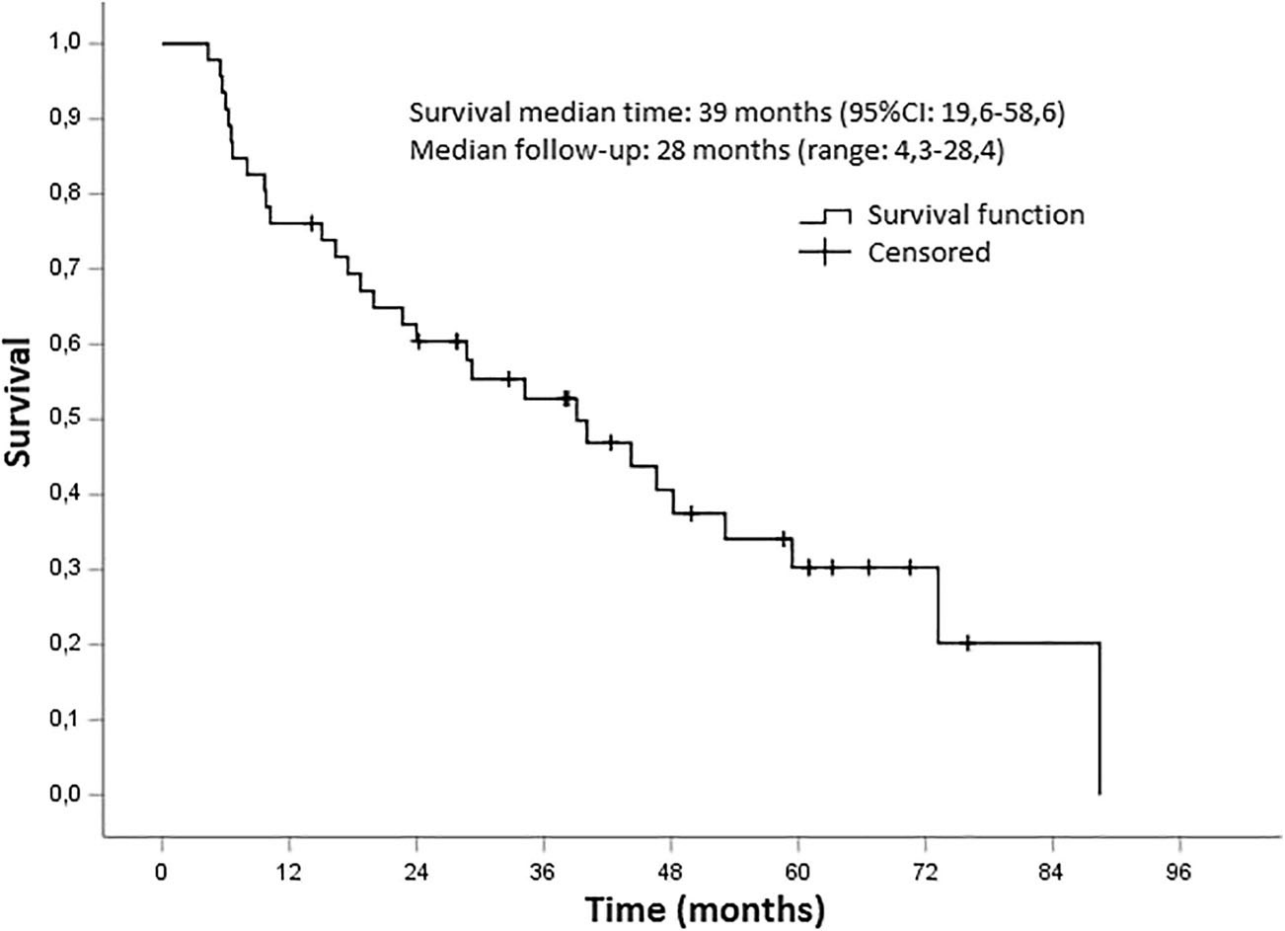
Systemic therapy-free survival curve

In-field progression was observed in 3 lesions. After the detection of oligorecurrent disease following SBRT, 6 patients underwent further courses of SBRT on the active lesions revealed by [^18^F]FMCH-PET/CT (two, three, four, five courses respectively in 4,1,1,1 patients). Toxicity higher than grade 2 was not recorded. At data analysis, median follow-up was 28.9 months (range 4.3–88.4), and systemic therapy was started in 24 patients (65.2%). Median systemic therapy-free survival was 39.1 months (95% CI 6.5–68.6, Fig. 1) whereas systemic free survival ratios at 6, 12, and 24 month were 93.5%, 73.9%, and 63.1%, respectively. Results of univariate analyses are reported in Table 2.

**Table 2:** Univariate analysis

	Univariate analysis				
Prognostic factor	RC	*p* value	HR	CI 95%	
GS	− 0.745	0.173	0.475	0.163	1.386
(0) 6; (1) 7-8-9					
Castration resistance	0.093	0.831	1.098	0.465	2592
(0) no; (1) sì					
Relapse	− 0.166	0.603	0.847	0.453	1.584
(0) Lfn; (1) bone; (2) both					
Number lesions	0.615	0.185	1.849	0.745	4.588
(0) 1; (1) > 1					
Delta PSA	3.394	< 0.001	29.77	6.875	> 50
(0) D; (1) U					
PSA DT	− 0.026	0.658	0.975	0.869	1.093

*RC*, regression coefficient

At univariate Cox regression analysis, Delta PSA demonstrated an impact on systemic therapy-free survival (*p* < 0.001).

## Discussion

To our knowledge, the present study represents one of the first prospective experiences carried out in patients with oligometastatic prostate cancer treated with SBRT. At the time of biochemical recurrence, all patients were re-staged using [^18^F]FMCH-PET/CT, and salvage treatment was selected based on the imaging findings. Patients with no more than 3 synchronous metastases were treated with repeated SBRT on all the detected lesions. Otherwise, systemic therapy was initiated. Since the prerequisite for a curative SBRT is an accurate patients’ selection, one of the most critical issues on oligometastatic patients with PCa is the accurate definition of the tumor burden, a critical aspect to planning local treatments [13]. In this study, we aimed to validate [^18^F]FMCH PET/CT as the technique of choice in selecting patients and active metastases to be treated with SBRT. The literature on this topic is failing, consisting of retrospective proof of concept studies; consequently, the importance of functional imaging in patients and target validation in oligometastatic PCa has not addressed yet. In 2013, Berkovic et al., in a retrospective analysis of 24 patients with oligometastatic PCa treated with SBRT after choline-PET/CT or [^18^F]FDG PET/CT assessment, reported a delay of anti-androgen therapy of 38 months and a 1-year and 2-year rate of ADT-free survival of 82% and 54%, respectively [8]. Similarly, Triggiani and Decaester obtained a median delay of anti-androgen therapy of 11 and 25 months, respectively [14, 15]. However, all these studies share the limitations of the lacking standard imaging technique, underestimating the importance of patients’ assessment before SBRT planning. A recent prospective randomized phase II trial by Ost et al. shows that metastasis-directed therapy (MDT) has advantages over surveillance in patients with up to three metastatic lesions from PCa, suggesting that MDT can be further explored in phase III trial to delay systemic therapy initiation [16]. Despite the prospective design, also in this study, the role of functional imaging in selecting patients suitable for SBRT was not deeply investigated. Indeed, we believe that the technique of patient selection would deserve a special focus, being the definition of oligometastatic state dependent on the imaging ability to detect macroscopic and microscopic disease. The emergence and widespread of whole body MRi and PSMA PET/CT have led to an increase detection of metastatic PCa, particularly at low PSA levels. [^68^Ga]PSMA-11 and [^18^F]-Flucicovine have shown improving PCa patients’ management and outcome. [^68^Ga]PSMA-11, despite not clinically approved, has outperformed standard-of-care imaging (including choline) for detecting PCa recurrence at low PSA values [17–19]. Indeed, a recent meta-analysis revealed detection rates with [^68^Ga]PSMA-11 PET/CT of 58% in patients with PSA between 0.2 and 1.0 ng/mL, 76% for PSA between 1 and 2 ng/mL and 95% for PSA > 2.0 ng/mL [20]. Nonetheless, its role in guiding radiation therapy even if promising is still to be validated [21], and very few data are available in the setting of oligometastatic disease [22, 23]. The use of [^18^F]Flucicovine resulted in a high positive predictive value for detecting extra prostatic disease in the setting of biochemical recurrence. However, it suffered from high false positive rates that could result in incorrectly upstaging the disease [24]. This will lead inevitably to a modification in the management of PCa. Therefore, the definition of oligometastatic disease should always be accompanied by the details of the imaging technique used for patients’ selection.

In the next future, we can foresee significant changes in the management of patients’ with recurrent PCa, as already anticipated by the inclusion in the latest EAU guideline of the use of PSMA-11 PET/CT (i) after RP if the PSA level is > 0.2 ng/mL and if the results will influence subsequent treatment decisions or (ii) after RT in patients fit for curative salvage treatment. We strongly believe that using [^68^Ga]PSMA-11 might significantly improve the detection of oligometastatic/oligorecurrent disease, as also sustained by the demonstration of [^68^Ga]PSMA-11 higher detection rate as compared with radiolabeled choline [25]. However, the present study was designed in the “pre [^68^Ga]PSMA-11 era” (i.e., 2010) when radiolabeled choline PET/CT was accounted for the most effective procedure in PCa and comparative data on a per-patients analysis of the two radiopharmaceuticals were not available. Indeed, despite choline-based radiopharmaceuticals might appear out-of-date, they still represent an elegant combination of clinical utility (good detection rate) and feasibility (registration and high availability for PCa restaging) as confirmed by the same EAU guideline in which the use of choline PET/CT (or fluciclovine PET/CT) is still recommended in case PSMA PET/CT is not available and the PSA level is > 1 ng/mL. Most important, we believe that the strength of the current study is related to its clinical message, independent from the radiopharmaceutical, which is the ability of SBRT in patients with oligometastatic disease of postponing the use of systemic therapy. In the next future, clinical trials using [^68^Ga]PSMA-11 as restaging procedure or comparing choline-based radiopharmaceuticals and [^68^Ga]PSMA-11 in patients with recurrent PCa are needed to demonstrate the impact of this new imaging modality on PCa patients’ outcome. Indeed, this is currently under investigation in our Center within a phase III prospective randomized clinical trial (Phase III clinical trial Oligometastatic and Oligorecurrent Prostate Cancer: enhancing patients’ selection by new imaging biomarkers – PROvING, EudraCT number 2018-004458-14).

Similarly, the definition of oligometastatic disease is evolving by moving from a definition based on limited number or limited location of the metastasis to the identification of biomarkers to segregate which patients should undergo to local treatment, thus by identifying key features of this biologically different entity compared with high-burden metastasis disease [26]. In the current study, we decided to apply the same definition of oligometastatic disease (up to three synchronous active lesions) as well the same SBRT schedules (24 Gy as a single fraction versus 27 Gy in three fractions) we already adopted in a prospective multicenter phase III clinical trial (NCT01223248) started in 2011 in patients with different type of cancer.

In our study, all patients were prospectively selected using the combination of pelvic MRI and [^18^F]FMCH PET/CT. Follow-up was based on PSA assessment, and in case of PSA rise further [^18^F]FMCH PET/CT was performed. By treating all the active lesions in 46 patients, we obtained a systemic therapy-free survival of 39.1 months (95% CI 6.5–68.6). The corresponding 6, 12, and 24-month systemic-free survival rate was 93.5%, 73.9%, and 63.1%, respectively. We used systemic therapy-free survival defined as the length from the PET/CT used to enroll patients in this study to the onset of at least four synchronous active metastases, and then the administration of systemic therapy, as a surrogate parameter of overall survival. This choice was based on the impossibility to use overall survival after [^18^F]FMCH-PET/CT-guided SBRT since at this stage of the clinical trial, all patients, beside one, are still alive. However, follow-up is still ongoing, and long-term results on overall survival are under evaluation. Through our preliminary results, we have already reported successful PCa patients’ selection for subsequent SBRT using [^18^F]FMCH-PET/CT [5]; the present paper confirms these preliminary findings in a larger number of patients with a longer follow-up. In our study, no significant difference between hormone naïve and castration-resistant patients was found. In fact, [^18^F]FMCH-PET/CT was able to identify oligometastatic patients not only at first biochemical recurrence but also after hormone therapy failure. Thus, it is in line with the results reported by Triggiani et al. in a multicenter retrospective study in oligometastatic castration-resistant patients treated with SBRT who found a median systemic treatment-free survival of 21.8 months and 1-year systemic treatment-free survival of 72.1% [27].

The median PSA value at the time of [^18^F]FMCH PET/CT was 2.69 ng/m. Univariate analysis showed that a fall of PSA value was associated with longer systemic therapy-free survival (*p* < 0.001). We interpreted this result as a consequence of a higher and longer response rate to SBRT in the presence of positive [^18^F]FMCH PET/CT, thus detecting all the sites of active disease and not only an ice cap.

[^18^F]FMCH-PET/CT has been investigated in the case of biochemical recurrence for patients’ selection for locoregional treatment such as salvage lymphadenectomy. Our findings on the use of functional imaging to guide local therapy in recurrent PCa are coherent with results reported in surgical series. In 2013, Tilki et al. reported results of 56 patients with PSA relapse treated with choline PET/CT-guided lymph node dissection. They identified at last one lymphatic metastasis in 85% of patients with positive PET/CT and only 4% of false positive in patients with PSA value higher than 2 ng/ml [28]. In 2015, Winter et al. reported their experience carried out in 13 patients, a biochemical response was observed in 11 out of 13 patients after an [^18^F]FMCH-PET/CT-guided lymphadenectomy. The authors concluded that their results could be improved in a larger series and prospective trials [29]. Moreover, Giovacchini et al., in a series of 358 biochemical PCa recurrent patients underwent radical prostatectomy, found a sensibility of 46% and 82% in patients with PSA level between 1 and 3 ng/mL and > 3, respectively [30].

In the present experience, toxicity higher than grade 2 was not recorded, and the safety we reported was the same observed in the previous studies [8, 15, 16]. The safety of SBRT is an essential consideration in the context of oligometastatic PCa patients who present longer life expectancy as compared with the plurimetastatic patients, therefore, making possible the onset of SBRT late toxicity. Based on our findings related to the safety of SBRT, and confirmed by other authors, we can be confident on the use of SBRT in oligometastatic PCa. Also, by postponing the administration of hormonal therapy or chemotherapy, we have also avoided the onset of potential toxicity, allowing a better quality of life that, in elderly patients with many comorbidities, can even affect overall survival.

## Conclusions

Based on our findings, [^18^F]FMCH PET/CT can identify oligometastatic patients suitable for SBRT, resulting in a systemic therapy-free survival of 39.1 months.

## References

[1] Mottet N, Bellmunt J, Bolla M, Briers E, Cumberbatch MG, De Santis M (2017). EAU-ESTRO-SIOG guidelines on prostate cancer. Part 1: screening, diagnosis, and local treatment with curative intent. Eur Urol.

[2] Cornford P, Bellmunt J, Bolla M, Briers E, De Santis M, Gross T (2017). EAU-ESTRO-SIOG guidelines on prostate cancer. Part II: treatment of relapsing, metastatic, and castration-resistant prostate cancer. Eur Urol.

[3] Mapelli P, Incerti E, Ceci F, Castellucci P, Fanti S, Picchio M (2016). 11C- or 18F-choline PET/CT for imaging evaluation of biochemical recurrence of prostate cancer. J Nucl Med.

[4] Evangelista L, Zattoni F, Guttilla A, Saladini G, Zattoni F, Colletti PM (2013). Choline PET or PET/CT and biochemical relapse of prostate cancer: a systematic review and meta-analysis. Clin Nucl Med.

[5] Pasqualetti F, Panichi M, Sainato A, Matteucci F, Galli L, Cocuzza P (2016). [(18)F]Choline PET/CT and stereotactic body radiotherapy on treatment decision making of oligometastatic prostate cancer patients: preliminary results. Radiat Oncol.

[6] Hellman S, Weichselbaum RR (1995). Oligometastases. J Clin Oncol.

[7] Pasqualetti F, Cocuzza P, Coraggio G, Ferrazza P, Derosa L, Galli L (2016). Long-term PSA control with repeated stereotactic body radiotherapy in a patient with oligometastatic castration-resistant prostate cancer. Oncol Res Treat.

[8] Berkovic P, De Meerleer G, Delrue L, Lambert B, Fonteyne V, Lumen N (2013). Salvage stereotactic body radiotherapy for patients with limited prostate cancer metastases: deferring androgen deprivation therapy. Clin Genitourin Cancer.

[9] Khoo V (2016). Is there another bite of the cherry? The case for radical local therapy for oligometastatic disease in prostate cancer. Eur Urol.

[10] Tree AC, Khoo VS, Eeles RA, Ahmed M, Dearnaley DP, Hawkins MA (2013). Stereotactic body radiotherapy for oligometastases. Lancet Oncol..

[11] Pasqualetti F, Panichi M, Sainato A, Baldaccini D, Cocuzza P, Gonnelli A (2018). Image-guided stereotactic body radiotherapy in metastatic prostate Cancer. Anticancer Res.

[12] Benedict SH, Yenice KM, Followill D, Galvin JM, Hinson W, Kavanagh B (2010). Stereotactic body radiation therapy: the report of AAPM Task Group 101. Med Phys.

[13] Franklin JM, Sharma RA, Harris AL, Gleeson FV (2016). Imaging oligometastatic cancer before local treatment. Lancet Oncol.

[14] Triggiani L, Alongi F, Buglione M, Detti B, Santoni R, Bruni A (2017). Efficacy of stereotactic body radiotherapy in oligorecurrent and in oligoprogressive prostate cancer: new evidence from a multicentric study. Br J Cancer.

[15] Decaestecker K, De Meerleer G, Lambert B, Delrue L, Fonteyne V, Claeys T (2014). Repeated stereotactic body radiotherapy for oligometastatic prostate cancer recurrence. Radiat Oncol.

[16] Ost P, Bossi A, Decaestecker K, De Meerleer G, Giannarini G, Karnes RJ (2015). Metastasis-directed therapy of regional and distant recurrences after curative treatment of prostate cancer: a systematic review of the literature. Eur Urol.

[17] Calais J, Czernin J, Fendler WP, Elashoff D, Nickols NG (2019). Randomized prospective phase III trial of (68)Ga-PSMA-11 PET/CT molecular imaging for prostate cancer salvage radiotherapy planning [PSMA-SRT]. BMC Cancer.

[18] Schwenck J, Rempp H, Reischl G, Kruck S, Stenzl A, Nikolaou K (2017). Comparison of (68)Ga-labelled PSMA-11 and (11)C-choline in the detection of prostate cancer metastases by PET/CT. Eur J Nucl Med Mol Imaging.

[19] Bluemel C, Krebs M, Polat B, Linke F, Eiber M, Samnick S (2016). 68Ga-PSMA-PET/CT in patients with biochemical prostate cancer recurrence and negative 18F-choline-PET/CT. Clin Nucl Med.

[20] Perera M, Papa N, Christidis D, Wetherell D, Hofman MS, Murphy DG (2016). Sensitivity, specificity, and predictors of positive. Eur Urol.

[21] Fendler WP, Eiber M, Beheshti M, Bomanji J, Ceci F, Cho S (2017). (68)Ga-PSMA PET/CT: joint EANM and SNMMI procedure guideline for prostate cancer imaging: version 1.0. Eur J Nucl Med Mol Imaging.

[22] Henkenberens C, von Klot CA, Ross TL, Bengel FM, Wester HJ, Merseburger AS (2016). Ga-PSMA ligand PET/CT-based radiotherapy in locally recurrent and recurrent oligometastatic prostate cancer: early efficacy after primary therapy. Strahlenther Onkol.

[23] Guler OC, Engels B, Onal C, Everaert H, Van den Begin R, Gevaert T (2018). The feasibility of prostate-specific membrane antigen positron emission tomography(PSMA PET/CT)-guided radiotherapy in oligometastatic prostate cancer patients. Clin Transl Oncol.

[24] Savir-Baruch B, Zanoni L, Schuster DM (2018). Imaging of prostate cancer using fluciclovine. Urol Clin North Am.

[25] Treglia G, Pereira Mestre R, Ferrari M, Bosetti DG, Pascale M, Oikonomou E (2019). Radiolabelled choline versus PSMA PET/CT in prostate cancer restaging: a meta-analysis. Am J Nucl Med Mol Imaging.

[26] Slaoui Amine, Albisinni S., Aoun F., Assenmacher G., Al Hajj Obeid W., Diamand R., Regragui S., Touzani A., Bakar A., Mesfioui A., Karmouni T., Ameur A., Elkhader K., Koutani A., Ibnattya A., Roumeguere T., Peltier A. (2019). A systematic review of contemporary management of oligometastatic prostate cancer: fighting a challenge or tilting at windmills?. World Journal of Urology.

[27] Triggiani Luca, Mazzola Rosario, Magrini Stefano Maria, Ingrosso Gianluca, Borghetti Paolo, Trippa Fabio, Lancia Andrea, Detti Beatrice, Francolini Giulio, Matrone Fabio, Bortolus Roberto, Fanetti Giuseppe, Maranzano Ernesto, Pasqualetti Francesco, Paiar Fabiola, Bonù Marco Lorenzo, Magli Alessandro, Bruni Alessio, Mazzeo Ercole, Franzese Ciro, Scorsetti Marta, Alongi Filippo, Jereczek-Fossa Barbara Alicja, Ost Piet, Buglione Michela (2019). Metastasis-directed stereotactic radiotherapy for oligoprogressive castration-resistant prostate cancer: a multicenter study. World Journal of Urology.

[28] Tilki D, Reich O, Graser A, Hacker M, Silchinger J, Becker AJ (2013). 18F-Fluoroethylcholine PET/CT identifies lymph node metastasis in patients with prostate-specific antigen failure after radical prostatectomy but underestimates its extent. Eur Urol.

[29] Winter A, Henke RP, Wawroschek F (2015). Targeted salvage lymphadenectomy in patients treated with radical prostatectomy with biochemical recurrence: complete biochemical response without adjuvant therapy in patients with low volume lymph node recurrence over a long-term follow-up. BMC Urol.

[30] Giovacchini G, Picchio M, Coradeschi E, Bettinardi V, Gianolli L, Scattoni V (2010). Predictive factors of [(11)C]choline PET/CT in patients with biochemical failure after radical prostatectomy. Eur J Nucl Med Mol Imaging.

